# Shock-Driven Endotheliopathy in Trauma Patients Is Associated with Leucocyte Derived Extracellular Vesicles

**DOI:** 10.3390/ijms232415990

**Published:** 2022-12-15

**Authors:** Romein W. G. Dujardin, Jeske E. C. Kisters, Mathijs R. Wirtz, Najat Hajji, Anita M. Tuip-de Boer, Jakob Stensballe, Pär I. Johansson, Karim Brohi, Ross A. Davenport, Christine Gaarder, Simon Stanworth, Marc Maegele, Rienk Nieuwland, Edwin van der Pol, Nicole P. Juffermans

**Affiliations:** 1 Laboratory of Experimental Intensive Care and Anesthesiology, Amsterdam UMC, University of Amsterdam, 1105 AZ Amsterdam, The Netherlands; 2 Department of Intensive Care Medicine, OLVG Hospital, 1091 AC Amsterdam, The Netherlands; 3 Department of Intensive Care Medicine, Rode Kruis Ziekenhuis, 1942 LE Beverwijk, The Netherlands; 4 Department of Biomedical Engineering and Physics, Amsterdam UMC, University of Amsterdam, 1105 AZ Amsterdam, The Netherlands; 5 Laboratory of Experimental Clinical Chemistry and Vesicle Observation Center, Amsterdam University Medical Center, University of Amsterdam, 1105 AZ Amsterdam, The Netherlands; 6 Department of Anesthesiology and Trauma Center, Center for Head and Orthopedics, and Section for Transfusion Medicine, Capital Region Blood Bank, Copenhagen University Hospital Rigshospitalet, 2100 Copenhagen, Denmark; 7 Trauma Sciences, Blizard Institute, Barts and the London School of Medicine and Dentistry, Queen Mary University of London, London E1 2AT, UK; 8 Department of Traumatology, Oslo University Hospital, 0372 Oslo, Norway; 9 NHS Blood and Transplant, Bristol BS34 7QH, UK; 10 Oxford University Hospital NHS Trust, John Radcliffe Hospital, Radcliffe Department of Medicine, University of Oxford, Oxford OX3 9DU, UK; 11 Department of Traumatology and Orthopedic Surgery, Cologne-Merheim Medical Center, University of Witten/Herdecke, 51109 Cologne, Germany

**Keywords:** endotheliopathy, extracellular vesicles, shock, trauma

## Abstract

Endotheliopathy following trauma is associated with poor outcome, but the underlying mechanisms are unknown. This study hypothesized that an increased extracellular vesicle (EV) concentration is associated with endotheliopathy after trauma and that red blood cell (RBC) transfusion could further enhance endotheliopathy. In this post hoc sub study of a multicentre observational trial, 75 trauma patients were stratified into three groups based on injury severity score or shock. In patient plasma obtained at hospital admission and after transfusion of four RBC transfusions, markers for endotheliopathy were measured and EVs were labelled with anti CD41 (platelet EVs), anti CD235a (red blood cell EVs), anti CD45 (leucocyte EVs), anti CD144 (endothelial EVs) or anti CD62e (activated endothelial EVs) and EV concentrations were measured with flow cytometry. Statistical analysis was performed by a Kruskall Wallis test with Bonferroni correction or Wilcoxon rank test for paired data. In patients with shock, syndecan-1 and von Willebrand Factor (vWF) were increased compared to patients without shock. Additionally, patients with shock had increased red blood cell EV and leucocyte EV concentrations compared to patients without shock. Endotheliopathy markers correlated with leucocyte EVs (ρ = 0.263, *p* = 0.023), but not with EVs derived from other cells. Injury severity score had no relation with EV release. RBC transfusion increased circulating red blood cell EVs but did not impact endotheliopathy. In conclusion, shock is (weakly) associated with EVs from leucocytes, suggesting an immune driven pathway mediated (at least in part) by shock.

## 1. Introduction

Trauma is an important cause of death worldwide, with severe haemorrhage being an important contributor to early death following trauma [1,2,3]. During the early phase of bleeding, trauma induced coagulopathy (TIC) can develop in response to tissue injury and shock. TIC is characterized by haemostatic dysfunction, resulting in enhanced bleeding which further contributes to organ failure and mortality [4].

The pathogenesis of TIC is multifactorial, but recent studies have demonstrated the importance of the endothelium in TIC. In a healthy state, the endothelium maintains vascular homeostasis by regulating inflammation and coagulation [5]. However, traumatic injury and the resultant haemorrhagic shock are associated with both endothelial glycocalyx degradation, resulting in syndecan-1 shedding and endothelial cell activation [6,7]. In turn, these processes trigger haemostatic dysfunction, leakage of the endothelial barrier, increased inflammation and organ failure, ultimately contributing to poor outcomes in trauma patients [8,9]. Accordingly, markers of endothelial dysfunction and glycocalyx degradation are directly associated with TIC and mortality [10,11]. Additionally, haemorrhaging trauma patients often receive blood products. Although transfusions are life-saving, they are also an independent predictor of mortality [12], which may, at least in part, be mediated by endothelial activation with ensuing haemostatic dysfunction, inflammation and organ failure. However, the mechanisms that drive the endothelium to shift from a healthy to an activated, pro-coagulant and pro-inflammatory state after trauma remain to be elucidated.

Extracellular vesicles (EVs) are circulating cell-membrane-derived particles that originate from a wide variety of cells, including platelets, leucocytes, red blood cells (RBC) and endothelial cells. EVs are released under both physiologic and pathologic conditions and are involved in intercellular communication, carrying surface markers, receptors and proteins from their cell of origin [13]. Recent evidence has demonstrated that plasma concentrations of EVs increase after trauma and that the injury severity score (ISS) is positively correlated with increased concentrations of both platelet-derived as well as endothelial-derived EVs [14]. In addition, EVs in RBC bags induce a strong and dose-dependent inflammatory host response in recipients [15]. Therefore, EVs may be a mediator of endothelial activation after trauma. As EV content can be modulated in blood products [16], the role of EVs in the host response to trauma is an important area of research. 

This study aims to investigate the association between EVs originating from different cell types and endotheliopathy in trauma patients. We hypothesize that severe injury with increased EV concentrations is associated with endothelial activation, which may be further enhanced by RBC transfusion. 

## 2. Results

### 2.1. Patient Characteristics and Outcome

The median age was 43 (27–57) years with a median ISS of 22 (10–30) points, 65 (87%) patients sustained a blunt trauma, 30 (40%) patients had traumatic brain injury and 11 (15%) patients were deceased at day 28 (baseline demographics in Table 1). Thirty patients (40%) presented with no shock, 26 (35%) with mild shock and 19 (25%) with severe shock. Patients with severe shock or a higher ISS had worse outcomes (Tables 1 and S1). Compared to patients that did not receive RBC transfusion, transfused patients had more severe shock, as reflected by lower systolic blood pressures in combination with a higher heart rate and increase in lactate (Table S2).

### 2.2. Endothelial Activation

**Figure 1:**
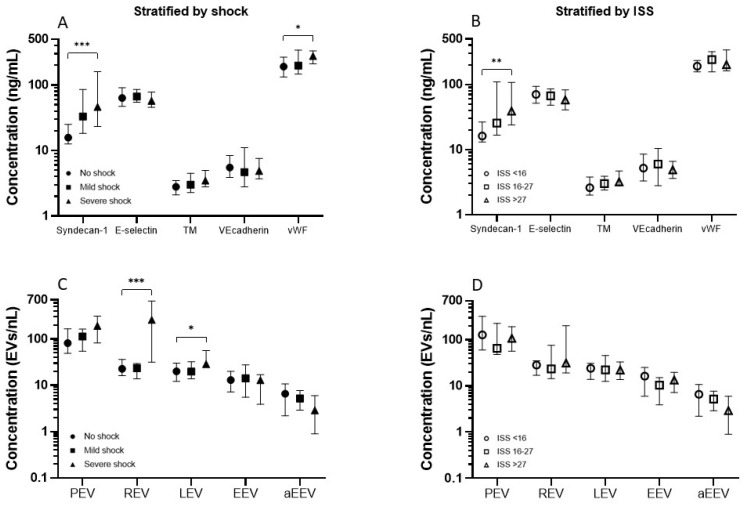
Median and interquartile ranges of (**A**) endothelial activation markers stratified by shock (**B**) endothelial markers stratified by injury severity score (ISS) and (**C**) Extracellular vesicles stratified by shock (**D**) extracellular vesicles stratified by ISS. A bar with asterisk represents a statistically significant difference between the groups, * *p* < 0.05, ** *p* < 0.01, *** *p* < 0.001. Abbreviations: TM: thrombomodulin, vWF: von Willebrand Factor, PEV: platelet derived extracellular vesicle, REV: red blood cell derived extracellular vesicle, LEV: leucocyte derived extracellular vesicles, EEV: endothelial derived extracellular vesicle, aEEV: activated endothelial derived extracellular vesicle.

In patients with severe shock, syndecan-1 and vWF concentrations were significantly increased compared to patients without shock (46.5 ng/mL vs. 15.8 ng/mL, *p* < 0.001 and 278.1 ng/mL vs. 191.6 ng/mL, *p* 0.034, respectively, Figure 1A). In the group of patients with an ISS > 27, only syndecan-1 concentrations were significantly increased compared to patients with an ISS < 16 (39.0 ng/mL vs. 16.2 ng/mL, *p* = 0.008, Figure 1B). Other endothelial markers did not differ between ISS groups. Regarding outcome, syndecan-1 concentrations were significantly higher in patients that died within 28 days compared to patients that survived (82.7 ng/mL vs. 23.2 ng/mL, *p* = 0.012).

### 2.3. Extracellular Vesicles

Patients with severe shock had increased red blood cell derived EV and leucocyte derived EV concentrations compared to patients without shock (259.5 EVs/nL vs. 22.8 EVs/nL, *p* < 0.001 and 28.7 EVs/nL vs. 20.0, *p* = 0.022, respectively, Figure 1C). There were no significant differences in the concentration of EVs between ISS groups (Figure 1D).

### 2.4. The Effect of Transfusion on Endothelial Activation and Extracellular Vesicles

**Figure 2:**
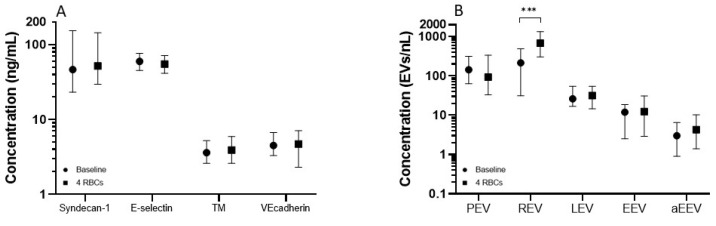
Median and interquartile ranges of levels of (**A**) endothelial activation markers on baseline and after transfusion and (**B**) Extracellular vesicles on baseline and after transfusion. A bar with asterisk represents a statistically significant difference, *** *p* < 0.001. Abbreviations: TM: thrombomodulin, PEV: platelet derived extracellular vesicle, REV: red blood cell derived extracellular vesicle, LEV: leucocyte derived extracellular vesicles, EEV: endothelial derived extracellular vesicle, aEEV: activated endothelial derived extracellular vesicle.

Transfused patients had significantly higher baseline concentrations of syndecan-1 and TM as well as platelet derived EVs, red blood cell derived EVs and leucocyte derived EVs than patients that did not require transfusion. There was no significant difference in endothelial activation marker concentrations before and after RBC transfusion (Figure 2A). RBC transfusions increased the concentrations of red blood cell derived EVs (683.1 EVs/nL vs. 215.0 EVs/nL, *p* < 0.001, Figure 2B), but not of other EVs.

### 2.5. The Relation between Endothelial Activation and Extracellular Vesicles

When EVs were correlated to syndecan-1 and vWF, only syndecan-1 correlated significantly with leucocyte derived EVs (ρ = 0.263, *p* = 0.023).

## 3. Discussion

This observational study suggests a shock-driven increase in syndecan-1 and vWF levels after trauma, with a correlation between syndecan-1 levels and leucocyte derived EVs, albeit weak. Although RBC transfusion was associated with increased red blood cell derived EVs, this did not result in enhanced endothelial activation.

Prior research has noted the importance of endotheliopathy in the outcome of trauma [10,11,17]. Shedding of the endothelial glycocalyx disrupts endothelial junctions, resulting in both activation of coagulation and increased vascular permeability [11,18,19] and may thereby be a pivotal contributor to poor outcome. We confirm previous studies showing an increase in concentrations of syndecan-1, vWF and a trend for an increase in TM in patients with severe shock compared to patients with no shock, indicating glycocalyx shedding and endothelial activation. We expand these findings by showing that the effect of injury severity on endothelial activation is less pronounced, indeed indicating that shock rather than injury severity underlies development of endotheliopathy. 

We further show that shock, rather than injury severity, is also the main driver of increased levels of red blood cell and leucocyte derived EVs. In line with this, a previous study demonstrated differences in EV concentrations between injured and healthy individuals, but not between patients with major and minor injury [20]. However, our results are in contrast with work that showed a correlation between both platelet as well as endothelial derived EVs and injury severity [14]. A possible explanation for this difference could be that the only platelet activation marker that was significantly associated with injury severity was P selectin, a marker for both platelet and endothelial activation that is also involved in leucocyte recruitment, which was not measured in this study. 

Of the EVs measured, only the plasma concentrations of leucocyte derived EVs correlated significantly with syndecan-1, which may suggest an immune driven pathway of glycocalyx degradation following trauma. Although the correlation reported in this study is weak, probably reflecting the multifactorial etiology of endotheliopathy in trauma, it is relevant considering the pivotal role of syndecan-1 in trauma related endothelial activation and poor outcome. Of note, besides endothelial barrier integrity, syndecan-1 is also involved in inhibition of leucocyte adhesion to the activated endothelium, inhibition of cytokine activity and confinement of leucocyte infiltration to specific sites [21]. Immune dysregulation following trauma is an important complication, associated with sepsis and organ failure [22,23]. Injury induces an inflammatory reaction, whereby neutrophils are activated with alterations of surface receptors and subsequently migrate across the endothelium, enhancing inflammation and development of organ damage [24,25]. It has been suggested that differences in neutrophil phenotypes reflect the severity of the systemic inflammatory response and could be used as a predictive tool for the management and prognosis of trauma patients [26,27]. Accordingly, a direct relationship between metabolic acidosis and expression of a neutrophil surface receptor for endothelial adhesion and migration has been described [27]. Our data contribute to the understanding of the detrimental effects of the systemic inflammatory response after trauma by suggesting that leucocyte derived EVs may contribute to shedding of the glycocalyx. At present, it is unknown how leucocyte-derived EVs contribute to endotheliopathy. However, it has been demonstrated that activated leucocytes release heparanase, which, upon activation, contributes to endotheliopathy by cleaving of syndecan-1. Owing to this, it is conceivable that leucocyte derived EVs also carry heparanase as cargo, which may explain the underlying mechanisms of leucocyte-derived EV endothelial activation as found in this study [28]. 

RBC transfusion resulted in an increase in red blood cell derived EVs, as shown before, but this did not correlate with any endothelial marker, nor did transfusion of red blood products impact EVs from other sources. This finding suggests that endogenous EV release is not triggered by exogenous transfusion. Accordingly, washing of transfusion products with removal of EVs did not improve outcome in a rat model of trauma and multiple transfusion [29]. Of note, RBC units in this study were leucoreduced. Considering that EV cargo is distinct between leucoreduced and non-leucoreduced units, results could be different when non-leucoreduced RBC units are used [30,31].

This study has limitations, including a small cohort, most likely resulting in insufficient power to demonstrate a significant increase in concentrations of TM and platelet derived EVs in the shock groups. Additionally, a sample selection bias could have been introduced to this study. Moreover, the pooling of patients with different injury types is a limitation. In addition, calculating correlations between multiple variables carries the risk of finding significant associations by chance, although we tried to limit this bias by only correlating EVs to endothelial markers that were significantly different between groups. Furthermore, flow cytometry for measuring EV concentrations has several limitations. By setting a gate, the lower detection limit of the flowcytometer was set at 200 ± 33 nm, which means that smaller EVs were not detected. The results therefore underestimate the actual EV concentration. Despite the analysis of a fraction of all EVs in plasma, significant variances in quantity and phenotype were found in this study. Additionally, pre-analytics and analytical factors, such as storage of EV samples, may have influenced results. To minimize the impact of analytical factors, the ISTH SSC guidelines for standardization of EV analysis [32] as well as the MIFlowCyt-EV reporting framework [33] were followed, thereby greatly enhancing reproducibility of our work. Lastly, due to the observational design of this study, causality cannot be inferred. 

In conclusion, after trauma, shock is a main driver of endotheliopathy and is (weakly) associated with increased leucocyte derived EVs. These results suggest an immune driven pathway of glycocalyx degradation following trauma, mediated (at least in part) by shock. Implications may be that modulating leucocyte immune response after trauma could impact outcome by conferring a protective effect on the endothelial glycocalyx.

## 4. Materials and Methods

### 4.1. Study Design and Participants

This study is performed as a post hoc analysis of the Activation of Coagulation and Inflammation in Trauma (ACIT) trial, a prospective observational multicentre study performed in 6 European level-1 trauma centres; London, Oslo, Copenhagen, Oxford, Cologne and Amsterdam, all members of the International Trauma Research Network (INTRN) [34]. Adult trauma patients (≥18 years) presenting with full trauma team activation were screened for inclusion. Patients were excluded if they received >2 L intravenous fluids before hospital admission, arrived at the emergency department >2 h after injury, suffered from burns covering >5% of the body or had pre-existing liver failure or bleeding disorders, were pregnant or taking oral anticoagulants other than aspirin. Transfusion protocols were consistent across all six sites, whereby transfusion was initiated in case of an arterial systolic blood pressure of < 90 mmHg in combination with a suspected bleed and limited or absent responsiveness to fluid resuscitation. RBC transfusions were leucoreduced in all sites. Informed consent was given by a personal consultee (e.g., a family member). When a patient regained physical and mental capacities, individual informed consent was obtained. This study was conducted in accordance with the Statement of the Declaration of Helsinki and performed after approval by the East London and The City Research Ethics Committee (07/Q0603/29) and the national ethics committee of participating centres. 

### 4.2. Patient Selection and Stratification

For this study, patients from the ACIT database were stratified into the following three predefined ISS groups: ISS < 16, ISS between 16 and 27 and ISS > 27 and matched on age and sex to control for these variables as confounders. Following stratification, 25 patients were chosen that received at least 4 RBC transfusions. Lastly, the remaining patients were balanced according to hospital to control for any centre effect, ultimately resulting in the 75 patients included for analysis. The same cohort of 75 patients was also stratified according to baseline base excess (BE) as a proxy of shock [35]: no shock (BE ≥ −2.00 mEq/L); mild shock (BE between −2.01 to −5.99 mEq/L); and severe shock (BE ≤−6.00 mEq/L).

### 4.3. Data Collection and Outcome Measures

Baseline demographics including mode of injury, ISS, vital signs, laboratory values at admission, prehospital fluid administration, amount of blood transfusions and 28-day mortality were collected to a centralized database. Blood samples drawn in citrated, heparin and EDTA tubes were collected within 20 min upon arrival at the emergency department and additional samples were collected after patients received 4 units of RBCs. The primary outcome was the concentration of endothelial activation markers and EVs. 

### 4.4. Sample Processing

Blood from trauma patients was drawn from an arterial catheter and within 20 min from blood collection, samples were centrifuged to obtain plasma. For enzyme linked immunosorbent assay (ELISA) measurements, samples were centrifuged for 10 min at 1750 RCF at 18 °C after which the upper 2/3 of plasma was divided equally over 3 eppendorfs and immediately stored at −80 °C until analysis. For flow cytometry, platelet depleted plasma was prepared by double centrifugation using a Rotina 46 RS equipped with a swing out rotor with a radius of 190 mm (Hettich Zentrifugen, Tuttlingen, Germany). Centrifugation parameters were 1750 RCF, 10 min, 18 °C, acceleration speed 9 and no brake. The upper two thirds of the plasma, consisting of a volume of 600 µL, was pipetted into a mixing tube and centrifuged again. After the second centrifuging step, the upper two thirds of the plasma was divided equally over 3 eppendorfs and stored at −80 °C until analysis. For EV analysis, samples were thawed at 37 °C and stored at room temperature, according to local protocol.

### 4.5. Endothelial Activation Markers

Levels of the following soluble endothelial markers at hospital arrival and after transfusion of 4 RBCs were determined with either enzyme linked immunosorbent assay (ELISA) or using an automated analyser: E-selectin (IBL International GMBH, Hamburg, Germany) with a lower limit of detection (LLD) of 0.3 ng/mL, Syndecan−1 (Diaclone Nordic Biosite, Copenhagen, Denmark) with an LLD of 4.94 ng/mL, thrombomodulin (TM, Nordic Biosite, Copenhagen, Denmark) with an LLD of 4.94 ng/mL, vascular endothelial cadherin (VEcadherin, R&D Systems Europe, Ltd., Abingdon, UK) with an LLD of 0.113 ng/mL and von Willebrand Factor (vWF) which was measured on an automated analyser (Sysmex CA-CS2100i, Siemens AG, Norderstedt, Germany). ELISAs were completed in duplicate and samples were measured again in case of an inter assay CV of >15%. Of note, levels of vWF after transfusion were not measured and therefore not available. 

### 4.6. Extracellular Vesicles and Flow Cytometry

Flow cytometry (A60-Micro, Apogee, Flow systems, Hertfordshire, UK) was performed in the Amsterdam University Medical Center (AUMC) to determine the concentration of EV subtypes in platelet-depleted plasma. We used a flow rate of 3.0 µL/min, measured each sample for 120 s, and triggered at side scattering. The reported concentrations described the number of particles: (a) that exceeded the side scatter threshold, which corresponds to a side scattering cross section of 10 nm^2^ (b) with a diameter >200 nm as determined by Flow-SR [36] (c) had a refractive index < 1.42 to omit false positively stained chylomicrons [37] and (d) that were positive at the fluorescence detector(s) corresponding to the used label(s) per mL of platelet-depleted plasma. EVs were labelled with anti-CD41-PE (Biocytex, Marseille, France), anti-CD235A-PE (Biolegend, San Diego, CA, USA), anti-CD45-APC (Biolegend, San Diego, CA, USA), anti-CD144-APC (Invitrogen, Waltham, MA, USA) or anti-CD62e-PE (BD, Franklin Lakes, NJ, USA) and characterized as platelet derived EVs (PEVs), red blood cell derived EVs (REVs), leucocyte derived EVs (LEVs), endothelial cell derived EVs (EEVs) and activated endothelial cell derived EVs (aEEVs), respectively. Samples from the transfusion group at baseline and after 4 RBC transfusions were analysed on the same well plate, to reduce variability. To validate test results, isotype controls (IgG1-PE or IgG1-APC, BD, Franklin Lakes, NJ, USA) were performed as well as buffer-only controls, buffer with reagents controls and unstained controls. All samples were measured using an autosampler, which facilitates subsequent measurements of samples in a 96-well plate. The entire study involved sixteen 96-well plates that were measured between 20 May 2019 and 2 October 2019. To automatically determine optimal samples dilutions, apply calibrations, determine and apply gates, generate reports with scatter plots and generate data summaries, we used custom-built software (MATLAB R2020b, Mathworks, Natick, MA, USA). All relevant details about sample preparation, assay controls, instrument calibration, data acquisition, and EV characterization are included in the supplemented reporting framework for the standardized reporting of EV flow cytometry experiments (MIFlowCyt-EV reporting guideline in Supplementary Material). This includes information on calibration of the fluorescence detectors from arbitrary units to molecules of equivalent soluble fluorochrome (Figure S1), forward scatter and side scatter calibration of the A60-micro by Rosetta calibration (Figure S2) an overview of staining reagents (Table S3) and MIFlowCyt-EV checklist (Table S4).

### 4.7. Statistical Analysis

Considering the hypothesis generating nature of this study, no a priori statistical power calculation was conducted. Distribution of data was assessed by visual inspection of histograms. Data is presented as median with interquartile range for numerical variables and as percentages for categorical variables. Continuous outcome variables were compared between ISS and shock groups using a Kruskall Wallis test adjusted by Bonferroni correction for multiple testing and a Mann–Whitney U test to compare between patients with and without transfusion. Categorical data was analysed with a Chi square test. The over-time comparison of endothelial markers and EVs at baseline and after 4 RBC transfusions was performed using a Wilcoxon signed rank test. Correlations between endothelial markers and EVs were calculated using a Spearman’s rho test. A two-tailed *p* value of <0.05 was considered statistically significant. Statistical analysis was performed with SPSS version 26.0 (IBM, New York City, NY, USA) and graphs were made with Graphpad version 9.1.0 (San Diego, CA, USA)

## Figures and Tables

**Table 1:** Baseline demographics of the whole cohort and patients stratified according to shock severity.

	Whole Cohort N = 75	No Shock N = 30	Mild Shock N = 26	Severe Shock N = 19	*p* Value
Age (years)	43 (27–57)	50 (35–62)	32 (21–48)	43 (31–58)	0.023
Male n (%)	54 (72%)	23 (77%)	19 (73%)	12 (63%)	0.584
Blunt injury n (%)	65 (87%)	28 (93%)	24 (92%)	13 (70%)	0.025
Coagulopathic n (%)	20 (27%)	4 (13%)	7 (27%)	9 (47%)	0.008
Transfusion group n (%)	25 (33%)	3 (10%)	5 (19%)	17 (90%)	<0.001
*Clinical Variables*					
ISS (0–75)	22 (10–30)	10 (8–23)	26 (16–39)	27 (22–33)	<0.001
TBI n (%)	30 (40%)	9 (30%)	11 (42%)	10 (53%)	0.321
Polytrauma n (%)	29 (38.7%)	5 (16.7%)	13 (30.0%)	11 (57.9%)	0.005
GCS (1–15)	13 (5–15)	15 (12–15)	9 (4–15)	10 (3–13)	0.035
SBP (mmHg)	127 (103–143)	141 (130–151)	123 (71–105)	94 (70–114)	<0.001
HR (beats/min)	83 (75–106)	80 (73–89)	80 (71–105)	120 (90–142)	<0.001
Mortality at day 28 n (%)	11 (15%)	0 (0%)	6 (23%)	5 (26%)	0.013
*Biochemical variables*					
Lactate (mmol/L)	2.6 (1.6–3.8)	2.1 (1.5–2.8)	2.1 (1.4–3.8)	4.6 (3.6–8.4)	<0.001
Base excess (mEq/L)	−2.6 (−6.2 to −0.8)	−0.25 (−1.3 to 1.1)	−3.8 (−4.5 to −2.5)	−9.6 (−15.5 to −7.4)	<0.001
Haemoglobin (mmol/L)	13.1 (12.0–15.3)	13.8 (13.0–14.5)	12.8 (11.8–14.0)	13.0 (11.4–15.0)	0.127
Platelet count (×10^9^/L)	223 (185–271)	244 (190–278)	234 (185–264)	214 (136–290)	0.336
aPTT (seconds)	25 (23–29)	24 (22–26)	26 (23–30)	30 (24–44)	0.005
INR (ratio)	1.1 (1.0–1.2)	1.0 (0.9–1.1)	1.1 (1.0–1.2)	1.2 (1.1–1.3)	<0.001
Fibrinogen level (g/L)	2.2 (1.6–2.7)	2.6 (2.1–3.3)	2.1 (1.5–2.5)	1.8 (1.4–2.8)	0.006

Abbreviations: aPTT, activated partial thromboplastin time; coagulopathic, defined as INR ≥ 1,2; GCS, Glasgow coma scale; HR, heart rate; INR, international normalized ratio; ISS, injury severity score; MOI, mechanism of injury; SBP, systolic blood pressure; TBI, traumatic brain injury, defined as AIS > 2; polytrauma defined as AIS ≥ 3 for two or more different body regions.

## Data Availability

Raw data, data with standard units and a summary of all flow cytometry scatter plots and gates applied will be shared upon request.

## Supplementary Materials

The following supporting information can be downloaded at: https://www.mdpi.com/article/10.3390/ijms232415990/s1, Table S1: Baseline characteristics of patients stratified by Injury Severity Score, Table S2: Baseline characteristics of patients stratified by transfusion, The elaborate MIFlowCyt-EV reporting tool including Table S3: Overview of staining reagents, Table S4: MIFlowCyt-EV checklist, Figure S1: Calibration of the fluorescence detectors from arbitrary units to molecules of equivalent soluble fluorochrome and Figure S2: Forward scatter and side scatter calibration of the A60-micro by Rosetta calibration.
