# Supplementary material for: Shock-Driven Endotheliopathy in Trauma Patients Is Associated with Leucocyte Derived Extracellular Vesicles
Source: Int J Mol Sci. 2022 Dec 15;23(24):15990. doi: 10.3390/ijms232415990 (PMC9782606; doi:10.3390/ijms232415990)
Supplement: Supplementary file 1 [file ijms-23-15990-s001.zip › ijms-2045315-supplementary.pdf]

International Journal of
Molecular Sciences

MDPI

Supplementary materials

Table S1. Baseline characteristics of patients stratified by Injury Severity Score (ISS).

	Whole Cohort	ISS <16	ISS 16-27	ISS >27	*p* Value
N	75	25	25	25	
Age (years)	43 (27–57)	38 (27–53)	43 (29–56)	47 (21–63)	0.840
Sex—males, n (%)	54 (72%)	20 (80%)	17 (68%)	17 (68%)	0.550
MOI—blunt, n (%)	65 (87%)	21 (84%)	19 (76%)	25 (100%)	0.030
Coagulopathic, n (%)	20 (27%)	2 (8%)	6 (24%)	12 (48%)	0.003
Transfusion group, n (%)	25 (33%)	3 (12%)	9 (36%)	13 (52%)	0.010
Clinical variables					
ISS score (0–75)	22 (10–30)	9 (5–10)	22 (17–25)	34 (30–45)	<0.001
TBI—yes n (%)	30 (40%)	2 (8%)	13 (52%)	15 (60%)	<0.001
Polytrauma n (%)	29 (38.7%)	0 (0%)	12 (48%)	17 (68%)	<0.001
GCS (1–15)	13 (5–15)	15 (13–15)	12 (9–15)	5 (3–13)	0.011
SBP (mmHg)	127 (103–143)	137 (114–147)	126 (100–151)	122 (90–134)	0.070
HR (beats/min)	83 (75–106)	80 (74–91)	83 (70–120)	89 (77–114)	0.310
Mortality 28-day—n (%)	11 (15%)	0 (0%)	4 (16%)	7 (28%)	0.010
Biochemical variables					
Lactate (mmol/L)	2.6 (1.6–3.8)	2.4 (1.9–3.0)	3.6 (2.1–6.5)	2.0 (1.3–3.8)	0.030
Base Excess (mEq/L)	−2.6 (−6.2 to -0.8)	−1.3 (−2.4 to 0.3)	−4.8 (−10.0 to −1.9)	−4 (−7 to −2)	0.001
Hemoglobin (mmol/L)	13.1 (12.0–15.3)	13.8 (12.9–14.5)	13.5 (11.9–14.8)	12.8 (11.6–13.4)	0.100
Platelet count ($\times 10^9$/L)	233 (185–271)	233 (189–267)	244 (176–288)	218 (169–252)	0.260
aPTT (seconds)	25 (23–29)	23 (22–25)	28 (23-31)	26 (24-30)	0.010
INR (ratio)	1.1 (1.0–1.2)	1.0 (0.9–1.1)	1.0 (0.9–1.1)	1.2 (1.0–1.3)	0.010
Fg level (g/L)	2.2 (1.6–2.7)	2.6 (2.2–3.1)	2.2 (1.6–2.7)	1.6 (1.3–2.2)	0.001

Abbreviations: aPTT, activated partial thromboplastin time; coagulopathic, defined as INR ≥1,2; Fg level, fibrinogen level; GCS, Glasgow coma scale; HR, heart rate; INR, international normalized ratio; ISS, injury severity score; MOI, mechanism of injury; SBP, systolic blood pressure; TBI, traumatic brain injury, defined as AIS>2; Polytrauma defined as AIS≥3 of two or more body regions. *p* value < 0.05 depicts a statistically significant difference between the three groups without post hoc testing.

Table S2. Baseline characteristics of patients stratified by transfusion.

	Non-Transfusion Group	Transfusion Group	*p* Value
N	50	25	
Age (years)	42 (26–54)	45 (30–59)	0.732
Sex—males n (%)	36 (72%)	18 (72%)	0.602
MOI—blunt n (%)	46 (92%)	19 (76%)	0.075
Coagulopathic n (%)	7 (14%)	13 (52%)	<0.001
Clinical variables			
ISS score (0–75)	17 (9–27)	29 (24–40)	<0.001
TBI—yes n (%)	18 (36%)	12 (48%)	0.251
Polytrauma n (%)	13 (26%)	16 (64%)	<0.001
GCS (1–15)	14 (6–15)	11 (3–14)	0.241
SBP (mmHg)	139 (123–145)	90 (72–112)	<0.001
HR (beats/min)	80 (71–90)	111 (81–138)	<0.001

Mortality 28-day n (%)	6 (12%)	5 (20%)	0.490
Biochemical variables			
Lactate (mmol/L)	2.2 (1.4–2.9)	3.8 (2.2–6.9)	<0.001
Base Excess (mEq/L)	−1.6 (−3.9 tot 0.5)	−7.6 (−10.8 to −4.3)	<0.001
Hemoglobin (mmol/L)	13.7 (12.5–14.4)	13 (12–14)	0.126
Platelet count ($\times 10^9$/L)	234 (187–271)	216 (156–285)	0.362
aPTT (second)	25 (23–29)	25 (23–35)	0.653
INR (ratio)	1.0 (0.9–1.1)	1.2 (1.1–1.2)	<0.001
Fg level (g/L)	2.5 (2.0–2.8)	1.6 (1.3–2.2)	0.003

Abbreviations: aPTT, activated partial thromboplastin time; coagulopathic, defined as INR ≥1,2; FFP, fresh frozen plasma; Fg level, fibrinogen level; GCS, Glasgow coma scale; HR, heart rate; INR, international normalized ratio; ISS, injury severity score; MOI, mechanism of injury; RBC, red blood cells; SBP, systolic blood pressure; TBI, traumatic brain injury, defined as AIS > 2, polytrauma defined as AIS≥3 of two or more body regions.

Table S3. Overview of staining reagents. Characteristics being measured, analyte, analyte detector, reporter, isotype, clone, concentration, manufacturer, catalog number and lot number of used staining reagents. The antibody concentration during measurements was 11.1-fold lower than the antibody concentration during staining.

Characteristic Measured	Analyte	Analyte Detector	Reporter	Isotype	Clone	Concentration During Staining (μg mL^{-1})	Manufacturer	Catalog Number	Lot Number
Integrin	Human CD41	Anti-human CD41 antibody	PE	IgG1	PL2-49	7.5	Biocyt	5112-PE100T	091251
Adhesion molecule	Human CD235a	Anti-human CD235a antibody	PE	**IgG1**	JC159	12.5	Dako Agilent	R7078	41236187
Leukocyte common antigen	CD45	Anti-human CD45 antibody	APC	IgG1	HI30	2.25	Biolegend	304037	B311564
Cadherin	CD144	Anti-human CD144 antibody	APC	IgG1	16B1	25	eBioscience	17-1449-42	4284172
Adhesion molecule	CD62e	Anit-human CD62e	PE	IgG1	68-5H11	12.5	Beckman Dickinson	551145	3038605
Affinity for Fc receptor	Fc receptor	IgG1	APC	n.a.	MPOC-21	25	Beckman Dickinson	554681	8261691
	Fc receptor	IgG1	PE	n.a.	X40	12.5	Beckman Dickinson	345816	9309643

APC: allophycocyanin; CD: cluster of differentiation; IgG: immunoglobulin G; PE: phycoerythrin.

Table S4. MIFlowCyt checklist.

Requirement	Please Include Requested Information
1.1. Purpose	See section 1.1.
1.2. Keywords	Endotheliopathy, extracellular vesicles, shock, trauma
1.3. Experiment variables	The severity of shock (based on baseline base excess) and injury severity score of trauma patients. Administration of red blood cell transfusion.
1.4. Organization name and address	Amsterdam University Medical Centers<br>Location Academic Medical Centre<br>Meibergdreef 9<br>1105 AZ Amsterdam

	The Netherlands
1.5. Primary contact name and email address	Romein W.G. Dujardin, r.w.dujardin@amsterdamumc.nl
1.6. Date or time period of experiment	May 2019 to October 2019
1.7. Conclusions	Patients with severe shock had increased red blood cell derived extracellular vesicles (EVs) and leucocyte derived EV concentrations compared to patients without shock (259.5 nL^{-1} vs. 22.8 nL^{-1}, $p < 0.001$ and 28.7 nL^{-1} vs. 20.0 nL^{-1}, $p = 0.022$, respectively). There were no significant differences in the concentration of EVs between injury severity score groups. In addition, RBC transfusion increased the concentrations of RBC-derived EVs, but not of other EVs.
1.8. Quality control measures	All samples were measured using an autosampler, which facilitates subsequent measurements of samples in a 96-well plate (see MIFlowCyt 1.9). Each well plate contained buffer-only controls (section S1.4), antibody in buffer controls (section S1.5), and isotype controls (section S1.7). The flow rate was checked with ApoCal beads and software (Apogee Flow Systems). Fluorescence detectors were calibrated (section S1.10) with 2 μm Q-APC beads (2321-175, BD) and SPHERO Easy Calibration Fluorescent Particles (ECFP-F2-5K, Spherotech Inc., Irma Lee Circle, IL, USA). FSC and SSC were calibrated with Rosetta Calibration (v1.29, section S1.11).
1.9 Other relevant experiment information	The entire study involved sixteen 96-well plates that were measured during 6 months.
2.1.1.1. Sample description	Thawed PDP (MIFlowCyt 2.1.1.2) from trauma patients (MIFlowCyt 2.1.1.3).
2.1.1.2. Biological sample source description	Blood from included trauma patients was drawn from an arterial catheter that was inserted upon emergency department arrival and collected in 2.7 mL citrated tubes (BD vacutainer, Plymouth). The first tube was discarded to avoid pre-activation of platelets. Within 20 minutes from blood collection, PDP was prepared by double centrifugation. Supernatant PDP was transferred into 1.5 mL Eppendorfs (Thermo Fisher Scientific) tubes, and stored in -80°C until analysis.
2.1.1.3. Biological sample source organism description	Hospitalized humans after trauma (for inclusion criteria, please see "Study design and participants" in the manuscript).
2.2 Sample characteristics	Within the detection range of our flow cytometer, PDP is expected to contain erythrocyte ghosts, EVs, chylomicrons, protein complexes, and platelets.
2.3. Sample treatment description	Please see section S1.3.
2.4. Fluorescence reagent(s) description	Please see Table S3.
3.1. Instrument manufacturer	Apogee, Hemel Hempstead, UK
3.2. Instrument model	A60-Micro
3.3. Instrument configuration and settings	Samples were analysed for 120 seconds at a flow rate of 3.01 μL/min on an A60-Micro, equipped with a 405 nm laser (100 mW), 488 nm laser (100 mW) and 638 nm laser (75 mW). The trigger threshold was set at SSC 14 arbitrary units, corresponding to a side scattering cross section of 10 nm^2 (Rosetta Calibration). For FSC and SSC, the PMT voltages were 380 V and 350 V, respectively. APC signals were collected with the 638-D Red(Peak) detector (long pass 652 nm filter, PMT voltage 510 V). PE signals were collected with the 488-Orange(Peak) detector (575/30 nm band pass filter, PMT voltage 520 V).
4.1. List-mode data files	Raw data, data with standard units and a summary of all flow cytometry scatter plots and gates applied are available upon request.
4.2. Compensation description	No compensation was required because single labelling was used.

4.3. Data transformation details	No data transforms were applied besides calibrations.
4.4.1. Gate description	See section 1.14.
4.4.2. Gate statistics	The number of positive events was corrected for flow rate, measurement time and dilutions performed during sample preparation.
4.4.3. Gate boundaries	On overview of all gates is available upon request.

CD: cluster of differentiation; EVs: extracellular vesicles; FSC: forward scattering; PDP: platelet free plasma; PMT: photo multiplier tube; SSC: side scattering.

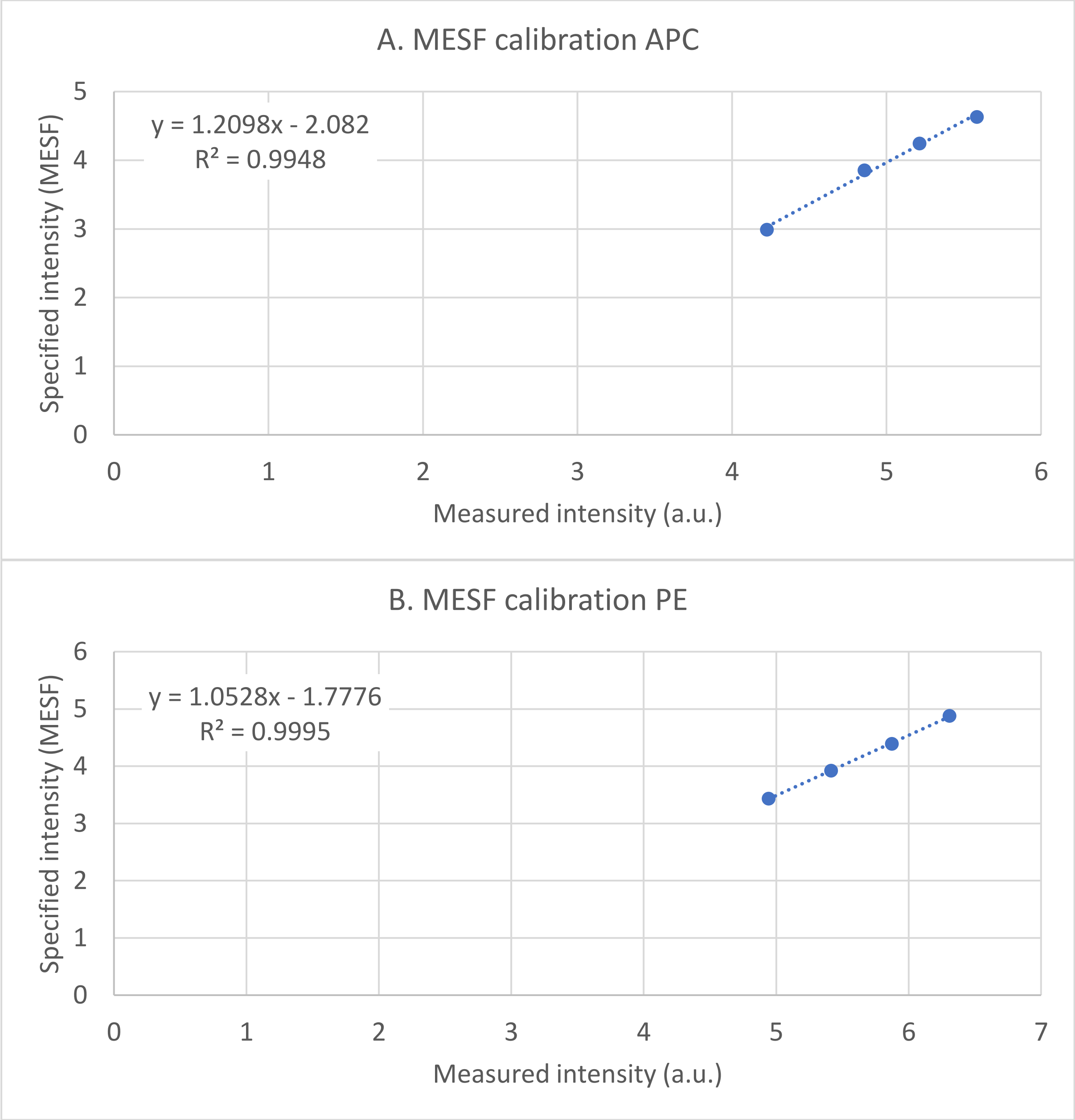

Figure S1. Calibration of the fluorescence detectors from arbitrary units (a.u.) to molecules of equivalent soluble fluorochrome (MESF). Logarithmic MESF versus logarithmic mean fluorescence intensity (MFI) for (**A**) APC, (**B**) phycoerythrin (PE). Data (symbols) are fitted with a linear function (line).

A. FSC

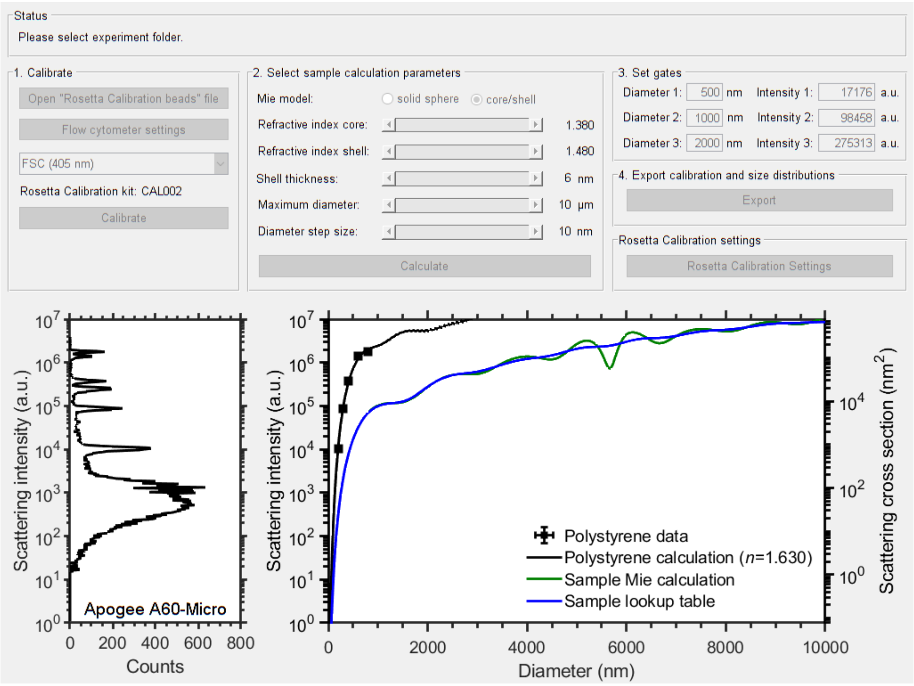

B. SSC

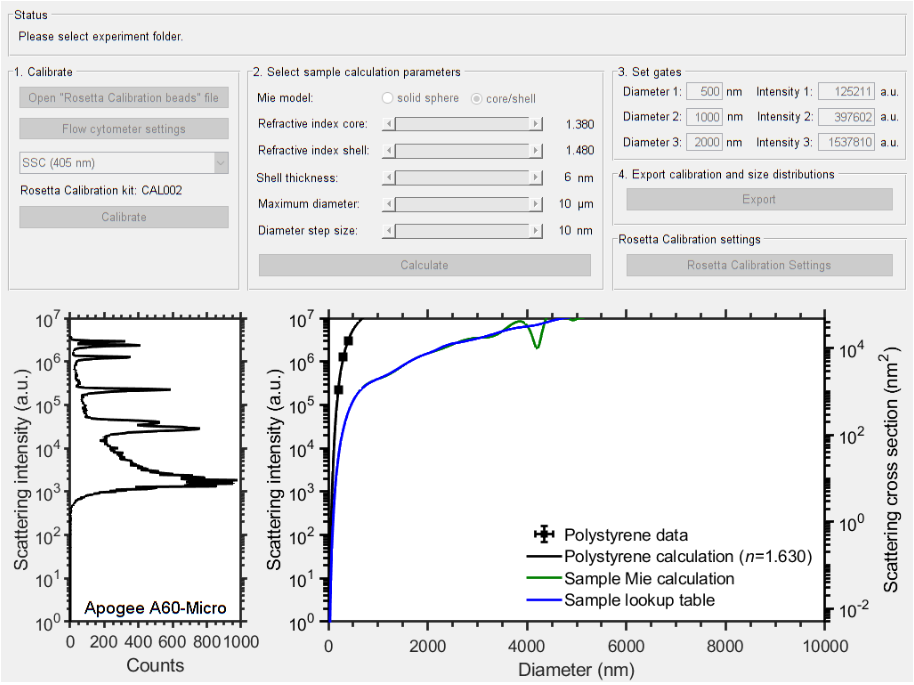

Figure S2. (**A**) Forward scatter and (**B**) side scatter calibration of the A60-Micro by Rosetta Calibration in 2018 and 2021. To relate scatter to the diameter of EVs, we modelled EVs as core-shell particles with a core refractive index of 1.38, a shell refractive index of 1.48, and a shell thickness of 6 nm.

MIFlowCyt-EV of clinical research study "Shock driven endotheliopathy in trauma patients is associated with leucocyte derived extracellular vesicles in trauma patients"

1. Flow cytometry

1.1. Experimental design

The aim of flow cytometry (A60-Micro, Apogee Flow Systems, Hemel Hempstead, UK) was to compare the concentrations of extracellular vesicles (EVs) released from platelets ($CD41^+$), red blood cells ($CD235a^+$), leukocytes ($CD45^+$), endothelial cells ($CD144^+$), and activated endothelial cells ($CD62E^+$) in platelet-depleted plasma (PDP) of 75 trauma patients with or without shock and of which 25 patients received at least 4 red blood cell transfusions.

We hypothesized that in patients with higher injury severity scores (ISS) after trauma, concentrations of red blood cell (RBC), platelet and endothelial derived extracellular vesicles (EVs) would increase and that this increase would be associated with increased concentrations of glycocalyx degradation products as well as increased levels of endothelial activation markers. Furthermore, we hypothesized that RBC transfusion would further increase concentrations of RBC-derived EVs and that this increase would further enhance endotheliopathy, visible as a correlation between concentration of RBC-derived EVs and the concentration of endothelial activation markers.

All samples were measured using an autosampler, which facilitates subsequent measurements of samples in a 96-well plate. The entire study involved sixteen 96-well plates that were measured between May 20th 2019 and October 2nd 2019. Each well plate contained buffer-only controls, antibody in buffer controls, and isotype controls. Flow rate and scatter calibrations were performed daily. To automatically determine optimal samples dilutions, apply calibrations, determine and apply gates, generate reports with scatter plots and generate data summaries, we used custom-built software (MATLAB R2020b, Mathworks, Natick, MA, USA).

1.2. Sample dilutions

As the particle concentration in PDP differs between individuals, samples require different dilutions to avoid swarm detection [1] and to achieve statistically significant counts within a clinically applicable measurement time. Although serial dilutions are recommended to find the optimal dilution, we consider serial dilutions unfeasible in a study with 75 donors. Therefore, we developed a procedure to estimate to optimal sample dilution (Section 1.2 of https://doi.org/10.6084/m9.figshare.c.4753676). In sum, we showed that for our flow cytometer and settings used, a count rate $\leq 5.0 \cdot 10^3$ events second unlikely results in swarm detection.

To find the dilution resulting in a count rate $\leq 5.0 \cdot 10^3$ events per second, we diluted each PDP sample 200-fold in Dulbecco phosphate buffered saline (DPBS) and measured the total concentration of particles for 30 seconds without staining. Samples having a count rate $> 5.0 \cdot 10^3$ following 200-fold dilution were diluted 2,000-fold and measured.

Taking into account the measured total concentration of particles and flow rate, we calculated the minimum dilution required before staining to achieve a count rate $\leq 5.0 \cdot 10^3$ events per second after staining. The staining procedure adds an extra dilution of 11.1-fold to the overall dilution.

1.3. EV staining

EVs in PDP were stained with antibodies. Prior to staining, antibodies were diluted in DPBS and centrifuged at 18,890 g for 5 min to remove aggregates. Table S3 shows an overview of the used reagents and antibody concentrations during staining. Each sample was single labelled with anti-CD41-PE (phycoerythrin), anti-CD235A-PE, anti-CD45-APC (allophycocyanin), anti-CD144-APC or anti-CD62e-PE. To stain, 20 μL of pre-staining diluted PDP was incubated with 2.5 μL of antibodies or isotype controls and kept in the dark for 2 h at room temperature. Post-staining, samples were diluted by adding 200 μL of DPBS to decrease background fluorescence from unbound reagents.

1.4. Buffer-only control

Each 96-wellplate contained at least 1 well with DPBS, which was measured with the same flow cytometer and acquisition settings as all other samples. The mean count rate was 36 events per second, which is substantially lower than the target count rate (2.5-$5.0 \cdot 10^3$ events per second) for PDP samples.

1.5. Buffer with reagents control

Each 96-wellplate contained a buffer with reagent control for each reagent (Table S3), which was measured with the same flow cytometer and acquisition settings as all other samples. Anti-CD41-PE, anti-CD45-APC, anti-CD62e-PE, and anti-CD144-APC in buffer resulted on average in 29, 18, 34, 31 events per second, respectively, and negligible fluorescence positive events. Anti-CD235a-PE in buffer resulted on average in 178 events per second. Despite the high total count rate of anti-CD235a-PE in buffer compared to the other antibodies in buffer, on average anti-CD235a-PE in buffer resulted in 34 positive counts per measurement, which is also negligible.

1.6. Unstained controls

Unstained controls were measured, but not used in our analysis.

1.7. Isotype controls

Table S3 shows an overview of the used isotype controls. Isotype control antibodies in DPBS resulted on average in 56 events per second and negligible fluorescence positive events.

1.8. Trigger channel and threshold

Based on the buffer-only control (36 events s^{-1}), the acquisition software was set up to trigger at 14 arbitrary units side scattering (SSC), which is equivalent to a side scattering cross section of 10 nm^2 (Rosetta Calibration, v1.29, Exometry, Amsterdam, The Netherlands).

1.9. Flow rate quantification

The A60-Micro is equipped with a syringe pump with volumetric control, which is checked on a daily basis using quality control beads and software (Apogee Flow Systems). In practice, the measured flow rate deviates at maximum 14% from the adjusted flow rate [2]. Therefore, we assumed that the flow rate is equal to the adjusted flow rate of 3.01 μL/min for all measurements.

1.10. Fluorescence calibration

Calibration of the fluorescence detectors from arbitrary units (a.u.) to molecules of equivalent soluble fluorochrome (MESF) was accomplished using 2 μm Q-APC beads (2321-175, BD) and SPHERO Easy Calibration Fluorescent Particles (ECFP-F2-5K, Spherotech Inc., Irma Lee Circle, IL, USA).

For each measurement, we added fluorescent intensities in MESF to the flow cytometry data files by custom-build software (MATLAB R2018a) using following equation:

$$\mathrm{I(MESF)} = 10^{a \cdot \log_{10} \mathrm{I(a.u.)} + b} \qquad \text{Equation S1}$$

where I is the fluorescence intensity, and *a* and *b* are the slope and the intercept of the linear fits in figures S1A-D, respectively.

1.11. Light scatter calibration

We used Rosetta Calibration to relate scatter measured by forward scattering (FSC) or SSC to the scattering cross section and diameter of EVs. Figure S2 shows print screens

of the scatter calibrations. We modelled EVs as core-shell particles with a core refractive index of 1.38, a shell refractive index of 1.48, and a shell thickness of 6 nm.

1.12. EV diameter and refractive index approximation

Flow-SR was applied to determine the size and refractive index of particles and improve specificity by enabling label-free differentiation between EVs and chylomicrons [3,4]. Flow-SR was performed as previously described [3,4]. Lookup tables were calculated for diameters ranging from 10 to 1000 nm, with step sizes of 1 nm, and refractive indices from 1.35 to 1.80 with step sizes of 0.001. The diameter and refractive index of each particle was added to the .fcs file by custom-build software (MATLAB R2020b).

As Flow-SR requires accurate measurements of both FSC and SSC, we applied Flow-SR only to particles with diameters >200 nm and fulfilling the condition:

$\mathrm{SSC}(\mathrm{nm}^2) > -0.7 \cdot \mathrm{FSC}(\mathrm{nm}^2) + 3.2$	Equation S2

1.13. MIFlowCyt checklist

The MIFlowCyt checklist is added to Table S4.

1.14. EV number concentration

The concentrations reported in the manuscript describe the number of particles (1) that exceeded the SSC threshold, corresponding to a side scattering cross section of 10 nm^2, (2) that were collected during time intervals, for which the count rate was within 750 events/s from the median count rate, (3) with a diameter >200 nm as determined by Flow-SR [3], (4) fulfilling the condition of equation S2, (5) having a refractive index <1.42 to omit false positively labeled chylomicrons, and (6) are positive at the corresponding fluorescence detector(s), per mL of PDP.

For the samples stained with CD41-PE, two extra gates were applied between aforementioned steps 2 and 3. To omit residual platelets, only events with a side scattering cross section <2,000 nm^2 and an PE intensity <7,000 MESF were included. To omit CD61 aggregates, only events fulfilling the condition:

$\mathrm{SSC}(\mathrm{nm}^2) > 1.0 \cdot \mathrm{APC}(\mathrm{MESF}) - 1.4$	Equation S3

were included.

1.15. Data sharing

Raw data, data with standard units, and a summary of all flow cytometry scatter plots and gates applied will be shared upon request.

References

1. van der Pol E, van Gemert MJC, Sturk A, Nieuwland R, van Leeuwen TG. Single vs. swarm detection of microparticles and exosomes by flow cytometry. *J Thromb Haemost* 2012; **10**: 919–30.
2. Gasecka A, Nieuwland R, Budnik M, Dignat-George F, Eyileten C, Harrison P, Lacroix R, Leroyer A, Opolski G, Pluta K, van der Pol E, Postuła M, Siljander P, Siller-Matula JM, Filipiak KJ, others. Ticagrelor attenuates the increase of extracellular vesicle concentrations in plasma after acute myocardial infarction compared to clopidogrel. *J Thromb Haemost* 2020; **18**: 609–23.
3. van der Pol E, de Rond L, Coumans FAW, Gool EL, Böing AN, Sturk A, Nieuwland R, van Leeuwen TG. Absolute sizing and label-free identification of extracellular vesicles by flow cytometry. *Nanomed Nanotechnol Biol Med* 2018; **14**: 801–10.
4. de Rond L, Libregts SFWM, Rikkert LG, Hau CM, van der Pol E, Nieuwland R, van Leeuwen TG, Coumans FAW. Refractive index to evaluate staining specificity of extracellular vesicles by flow cytometry. *J Extracell Vesicles* 2019; **8**: 1643671.
